# Impact of baseline cases of cough and fever on UK COVID-19 diagnostic testing rates: estimates from the Bug Watch community cohort study

**DOI:** 10.12688/wellcomeopenres.16304.2

**Published:** 2021-01-25

**Authors:** Max T. Eyre, Rachel Burns, Victoria Kirkby, Catherine Smith, Spiros Denaxas, Vincent Nguyen, Andrew Hayward, Laura Shallcross, Ellen Fragaszy, Robert W. Aldridge

**Affiliations:** 1 Centre of Health Informatics, Computing and Statistics, Lancaster Medical School, Lancaster University, Lancaster, LA1 4YW, UK; 2 Liverpool School of Tropical Medicine, Liverpool, L3 5QA, UK; 3 Centre of Public Health Data Science, Institute of Health Informatics, University College London, London, NW1 2DA, UK; 4 Institute of Health Informatics, University College London, London, NW1 2DA, UK; 5 Health Data Research UK, London, NW1 2DA, UK; 6 The Alan Turing Institute, London, NW1 2DB, UK; 7 The National Institute for Health Research University College London Hospitals Biomedical Research Centre, University College London, London, W1T 7DN, UK; 8 British Heart Foundation Research Accelerator, University College London, London, NW1 2DA, UK; 9 Institute of Epidemiology and Health Care, University College London, London, WC1E 7HB, UK; 10 Faculty of Epidemiology and Population Health, London School of Hygiene and Tropical Medicine, London, WC1E 7HT, UK

**Keywords:** COVID-19, cough, fever, diagnostic testing capacity, United Kingdom, swab test

## Abstract

**Background: **Diagnostic testing forms a major part of the UK’s response to the current coronavirus disease 2019 (COVID-19) pandemic with tests offered to anyone with a continuous cough, high temperature or anosmia. Testing capacity must be sufficient during the winter respiratory season when levels of cough and fever are high due to non-COVID-19 causes. This study aims to make predictions about the contribution of baseline cough or fever to future testing demand in the UK.

**Methods: **In this analysis of the Bug Watch community cohort study, we estimated the incidence of cough or fever in England in 2018-2019. We then estimated the COVID-19 diagnostic testing rates required in the UK for baseline cough or fever cases for the period July 2020-June 2021. This was explored for different rates of the population requesting tests, four COVID-19 second wave scenarios and high and low baseline cough or fever incidence scenarios.

**Results: **Under the high baseline cough or fever scenario, incidence in the UK is expected to rise rapidly from 250,708 (95%CI 181,095 - 347,080) cases per day in September to a peak of 444,660 (95%CI 353,084 - 559,988) in December. If 80% of these cases request tests, testing demand would exceed 1.4 million tests per week for five consecutive months. Demand was significantly lower in the low cough or fever incidence scenario, with 129,115 (95%CI 111,596 - 151,679) tests per day in January 2021, compared to 340,921 (95%CI 276,039 - 424,491) tests per day in the higher incidence scenario.

**Conclusions: **Our results show that national COVID-19 testing demand is highly dependent on background cough or fever incidence. This study highlights that the UK’s response to the COVID-19 pandemic must ensure that a high proportion of people with symptoms request tests, and that testing capacity is sufficient to meet the high predicted demand.

## Introduction

In response to the spread of novel coronavirus severe acute respiratory syndrome coronavirus 2 (SARS-CoV-2), the United Kingdom (UK) has implemented large-scale public health measures that aim to reduce transmission and contact rates in the population. Maintaining control through diagnostic testing and self-isolation will increasingly depend upon self-diagnosis based on an individual’s symptoms. Current National Health Service (NHS) guidance is that any person in the community who develops at least one symptom of: a new continuous cough, a high temperature, or a loss of, or change in, normal sense of taste or smell (anosmia), should schedule a swab test with NHS services for home delivery or visit a testing site and self-isolate for up to 10 days after onset of symptoms or until a negative test result is received ^1– 3^. The NHS Test and Trace service aims to trace and notify close recent contacts of anyone who tests positive for coronavirus, instructing them to self-isolate for 14 days.

The importance of COVID-19 testing in the UK’s response to the current pandemic is apparent in the five-pillar testing strategy, described by the UK Government in April 2020. The first two pillars use swab-based testing and molecular diagnosis of COVID-19 using real-time PCR ^4^. Pillar 1 of the testing strategy relates to the swab testing for health and care workers and those with a clinical need, carried out by Public Health England (PHE) and NHS labs. Pillar 2 concerns swab testing for the wider population including social care and is carried out with commercial partners. The UK Government stated that an important marker for easing control measures and restrictions included having confidence that operational challenges, such as testing capacity, were “ *in hand, with supply able to meet future demand*” ^5^.

Fever and cough are common symptoms in other acute respiratory viruses ^6^. As a result of the non-specific nature of these respiratory symptoms, a large number of individuals meeting the UK’s COVID-19 diagnostic testing criteria – and being subsequently tested – will have cough and/or fever caused by a non-COVID-19 infection. It is therefore important to estimate the total number of cases in the population that would meet the diagnostic testing criteria (including both COVID-19 and non-COVID-19 cases) and the proportion of these cases that would seek testing, in order to ensure sufficient diagnostic testing capacity.

Bug Watch was a prospective community cohort study conducted in England in 2018–2019 that collected daily information on symptoms of a range of acute common infections ^7^. Data collected within the study allows us to estimate the community incidence of fever and cough symptoms in England and describe seasonal patterns across a calendar year. Our study has two main objectives: first, to use Bug Watch data to estimate the all-age monthly incidence of cough or fever in England in the period 2018–2019; second, to estimate the UK COVID-19 diagnostic testing demand under current government testing policy for July 2020 – June 2021.

## Methods

### Study design, recruitment and data collection

Bug Watch was an online prospective community cohort study in England. Full details of the study design, recruitment and data collection are described in the protocol ^7^. In brief, participants were recruited through an invitation letter sent to adults who participated in the 2013, 2014 and 2015 Health Survey for England (HSE). Parents or guardians were asked to register their children under 16 and complete surveys on their behalf. Any other adults within the same household were invited to register separately. Recruitment was conducted in four waves in March, June, September and November 2018. Data collected consisted of an online consent form and a baseline survey followed by weekly surveys sent by email to be completed by each participant. Each week, participants were asked to prospectively keep track of a wide range of symptoms of infection using a symptom diary. The primary outcomes of interest for this study were cough (defined as either a dry cough or coughing up phlegm) and fever. Each individual was followed up for six months. Only individuals with a 75% completion rate were included in the analysis.

Out of 19,741 adults who were invited to join the study, a total of 873 participants were included in the analysis (782 adults and 91 children that they had registered), providing a total follow-up time of 23,111 person-weeks. Cohort baseline characteristics have been described in more detail ^8^, and are included in Extended data, S1 ^9^. In terms of indicators of potential selection bias, participants were more likely to be older, female, healthier and living in less deprived areas than the general population of England. Age and sex were adjusted for in subsequent statistical analyses, but healthiness and deprivation levels may have skewed measured incidence rates in the cohort towards lower values than the general population.

### Ethics

Data were collected using Research Electronic Data Capture (REDCap)17 surveys hosted on the UCL Data Safe Haven, which is certified to the ISO27001 information security standard and conforms to NHS Digital’s Information Governance Toolkit. This study was given ethical approval by the UCL Research Ethics Committee (ID 11813/001).

### Statistical analysis

***Baseline incidence of cough or fever in England.*** The first ten days of follow-up after each participant was recruited into the study were excluded to remove prevalent infection syndromes. For participants reporting cough or fever symptoms within these first ten days, follow-up was started on the first day after this period with no symptoms. Incident cough or fever were defined as i) when a participant reported cough or fever for the first time (one day of symptoms was recorded as a case); or ii) when either symptom was reported after a period of at least 10 days without symptoms. Non-specific symptoms could extend the duration of a cough or fever infection period. Public Health England reported that there were “low to moderate levels of influenza activity” in the 2018–2019 influenza season, which was comparable to the 2017–2018 season and higher than all other seasons since 2010–2011 ^10^.

Monthly adjusted incidence rates per 100,000-person-week for cough or fever and confidence intervals were calculated for England, weighting to the mid-2019 population structure of England for age, sex and region ^11^ by post-stratification using a quasi-Poisson regression model (with the R ‘survey’ package version 4.0 ^12^). Monthly age-specific incidence rates per 100,000-person-week for cough or fever in England were calculated, weighting by sex and region, and are included in *Extended data,* S2 ^9^.

***UK testing demand due to baseline cough and fever cases.*** Monthly all-age adjusted incidence rates of baseline (non-COVID-19) cough or fever were estimated for the UK, weighting to the mid-2019 population structure of the UK for age and sex ^11^. These rates were used to estimate the average number of individuals in the UK with an incident case of non-COVID-19 cough or fever each day for each month in the period July 2020 – June 2021.

Predictions for the daily testing demand expected in the UK between July 2020 and June 2021 due to baseline cough or fever cases were made based on our incidence estimates. We assumed that individuals only request a test on the first day that they experience symptoms. We explored a range of scenarios for the proportion of cough or fever cases that request a test (PROPTEST). Four values were explored: 40%, 60%, 80% and 100%. The predicted impact of baseline cough or fever cases on UK testing capacity was calculated as the difference between UK Pillar 1 and 2 laboratory testing capacity estimates from August 2020 and predicted testing demand between July 2020 and June 2021 based on these scenarios. Capacity estimates used in this analysis were reported by the UK government for the period 6–12th August 2020 as 1,459,418 tests per week for Pillars 1 and 2, and 880,000 tests per week for only Pillar 2 ^13^.

***Total UK testing demand including symptomatic COVID-19 cases.*** Four scenarios (C1–C4) for additional demand due to a second COVID-19 wave in the UK during winter 2020–2021 were explored. A range of average daily incidences for COVID-19 cases for each month between July 2020 and June 2021 were considered to reflect uncertainty about future COVID-19 transmission levels, from the lowest in scenario C1 to the highest incidences in scenario C4. We used an exponentially weighted multiplication factor with minimum values of 0.002, 0.004, 0.006 and 0.008 in August for scenarios C1–C4, respectively, increasing to peak values of 0.05, 0.10, 0.15 and 0.20 in January – March for these four scenarios. This multiplication factor was then multiplied by the estimated daily incidence of cough or fever in the UK for each month to provide hypothetical daily COVID-19 incidences for each month. An extended description of these methods can be found in *Extended data,* S3 ^9^. These scenarios were selected to follow a similar epidemic curve shape to predictions reported in the Academy of Medical Sciences’ report “Preparing for a Challenging Winter 2020/21” ^14^, with the highest incidences in January and February 2021 and the peak incidence in our worst-case scenario (C4) equal to the peak incidence predicted in the report for a reproductive number R _t_=1.5 between September 2020 to July 2021. Based on estimates from the COVID Symptom Study app, 87.5% of these cases of COVID-19 were assumed to exhibit symptoms of cough, fever or loss of smell or taste ^15^ with the proportion of these symptomatic cases expected to request tests explored using the PROPTEST parameter. Total demand for swab tests due to baseline cough and fever cases and COVID-19 illnesses was calculated as:

TotalDemand=(baselinecoughorfeverincidence+COVID19incidence×0.875)×PROPTEST

The total predicted testing demand in each month was then calculated for each COVID-19 scenario (C1–C4).

***Total UK testing demand in a low cough or fever incidence scenario*.** Estimated total UK testing demand was adjusted to explore the impact of a reduced incidence of baseline cough or fever cases relative to previous years due to social distancing and other COVID-19 public health interventions. Estimates of the relative reduction in monthly baseline cough or fever incidence for July – November 2020 compared to the corresponding months in the 2018–2019 Bug Watch cohort were available from the preliminary results of the ongoing Virus Watch cohort study (see www.ucl-virus-watch.net for more information about the study) in England (unpublished report, author: Robert Aldridge). These found that the median reduction in non-COVID-19 cough or fever incidence in these months relative to the Bug Watch baseline was 73% (range 34% – 81%). This was used to recalculate estimates of the total UK testing demand for each month assuming a 73% lower incidence of cough or fever relative to our historical baseline for each month.

All analyses were conducted using R version 3.6.3 ^16^ using the R ‘ tidyverse’ packages version 1.3.0 ^17^.

## Results

### Cough or fever incidence in England

Out of a total of 585 episodes of cough or fever, participants experienced 431 (73.7%; 431/585) episodes of cough, 57 (9.7%; 57/585) of fever and 97 (16.6%; 97/585) episodes with both cough and fever symptoms.

**Figure 1.**
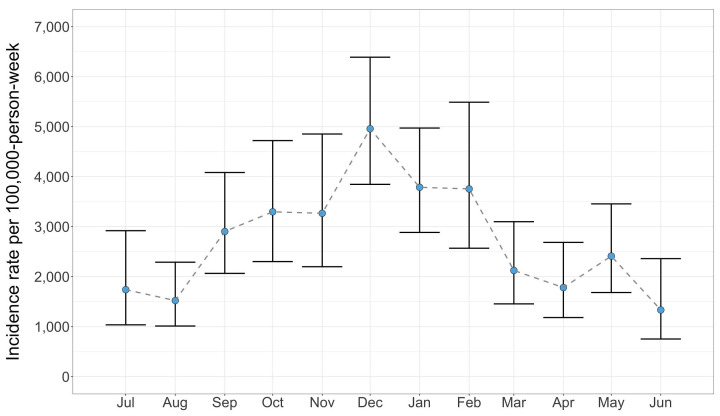
Monthly adjusted incidence rates of cough or fever per 100,000-person-week in England with 95% confidence intervals. Weighted to the mid-2019 population structure of England by age, sex and region.

Monthly age-, sex- and region-adjusted incidence rates of cough or fever per 100,000-person-week and 95% confidence intervals are shown for the 12-month study period in England in Figure 1. There was clear seasonal variation in incidence, with the lowest rates in June and highest rates in December with 1,333 (95%CI 753 – 2,361) and 4,958 (95%CI 3,847 – 6,390) incident episodes of cough or fever per 100,000-person-week, respectively. The high incidence in December coincides with UK public holidays and lower temperatures, when indoor contact rates are higher.

### UK testing demand due to baseline cough and fever cases

**Figure 2.**
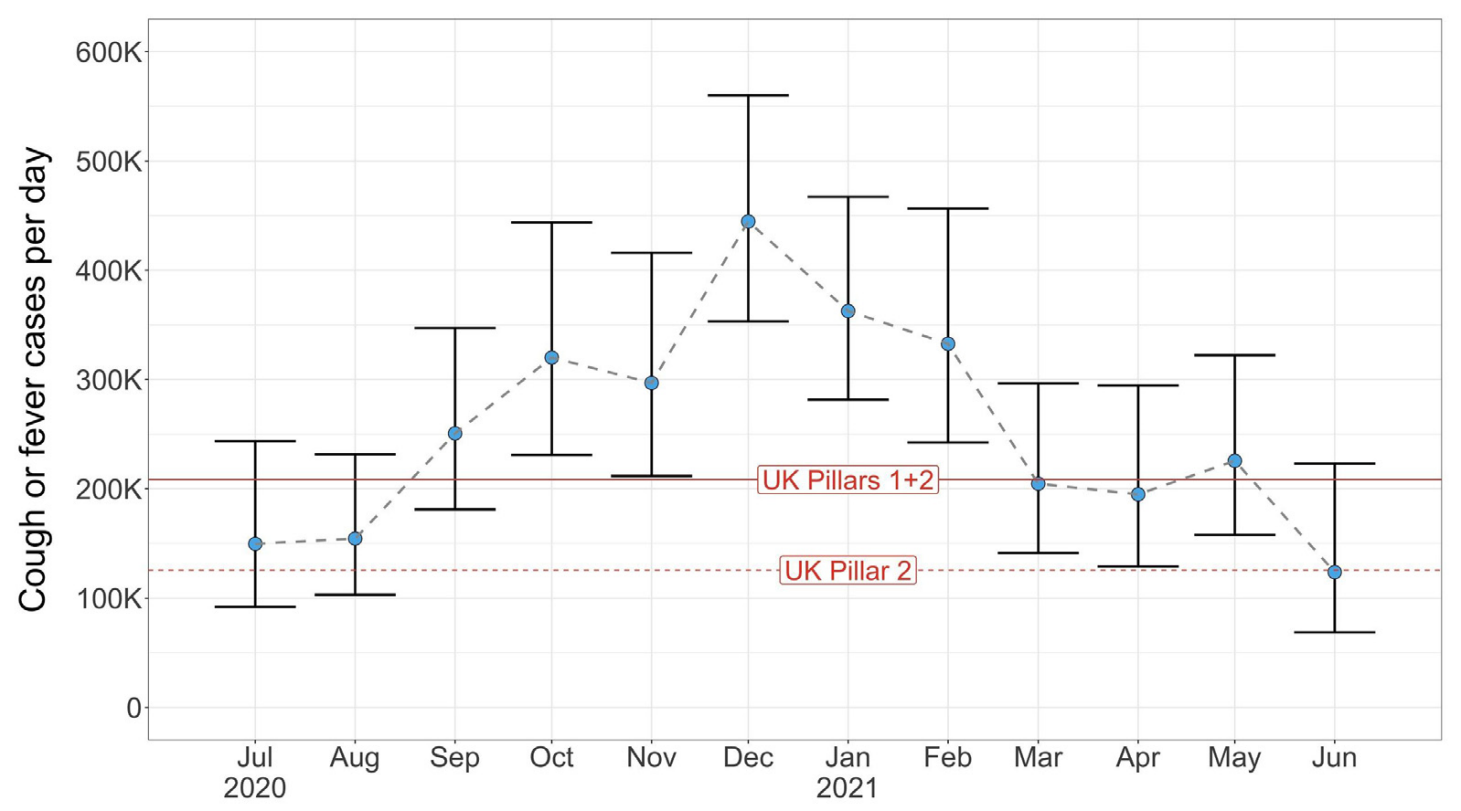
Predicted baseline number of individuals (in thousands) in the UK with an incident case of cough or fever each day shown for each month. Laboratory capacity (measured as daily tests) estimates from August 2020 for Pillars 1 and 2 and Pillar 2 in the UK are shown as solid and dashed red lines, respectively.

Predictions for the average daily number of baseline (non-COVID-19) cough or fever cases in the UK between July 2020 – June 2021 are shown for each month in Figure 2. Under current UK government policy, all of these cases would be entitled to a COVID-19 swab test. After the lower incidence summer period of 2020, the incidence starts to rise rapidly, increasing from 154,554 (95%CI 103,083 – 231,725) cases per day in August to 250,708 (95%CI 181,095 – 347,080) in September, before peaking at 444,660 (95%CI 353,084 – 559,988) daily cases in December. This high incidence continues to exceed UK laboratory testing capacity in August 2020 for Pillars 1 and 2 until the end of winter before falling to 204,750 (95%CI 141,392 – 296,499) cases per day in March 2021.

**Figure 3.**
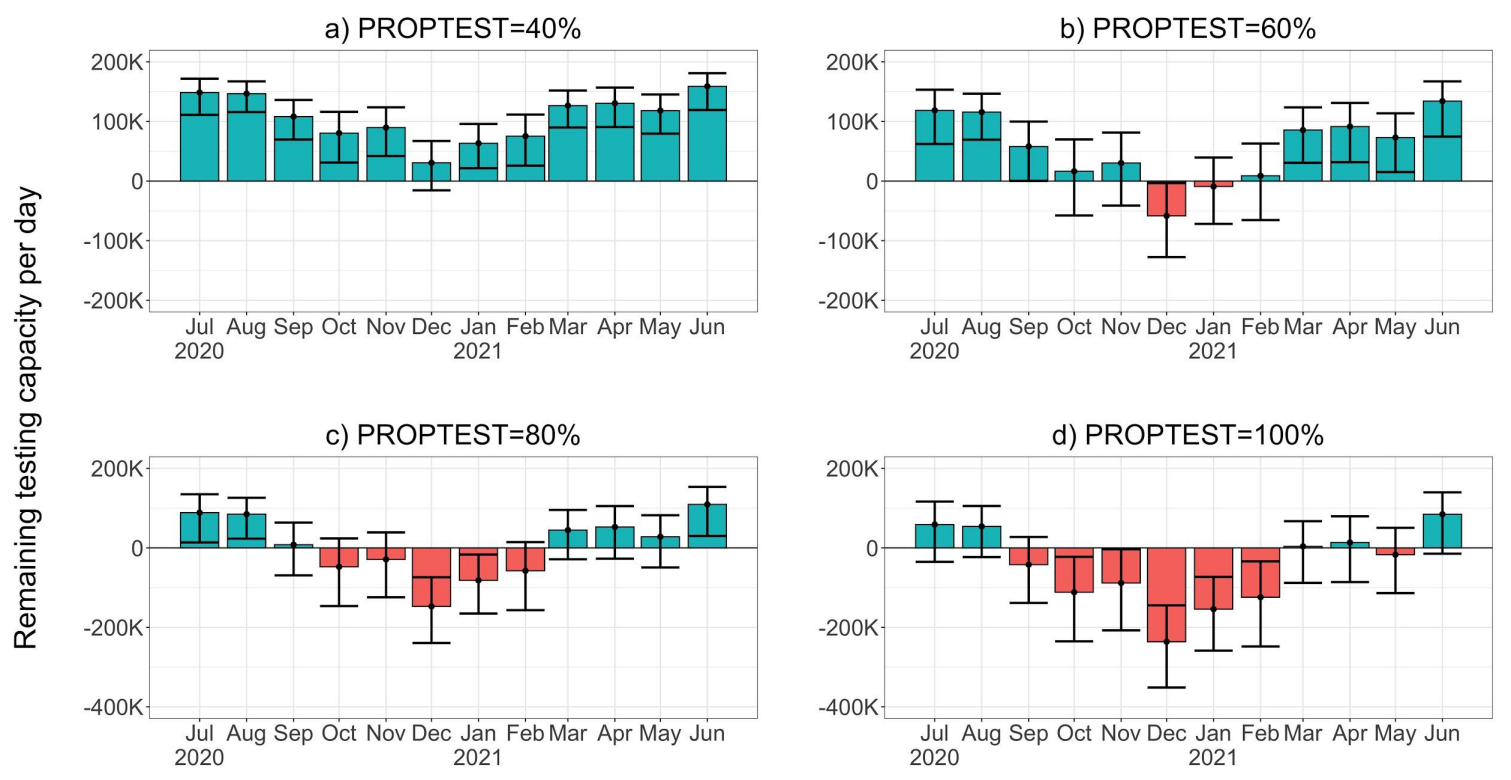
Remaining UK Pillar 1 and 2 testing capacity (thousands of tests) available after testing baseline (non-COVID-19) cases of cough or fever in the UK each day. Panels **a**) to **d**) show results for four values of the proportion of cough of fever cases requesting tests (PROPTEST). Blue and red bars indicate that cough or fever testing demand is within or in excess of capacity available in August 2020, respectively.

Remaining UK Pillar 1 and 2 capacity after testing baseline cough or fever cases is shown in Figure 3 for four values of the proportion of cough or fever cases which request tests (PROPTEST). The peak in cases in the autumn and winter of 2020–2021 is likely to place significant stress on the UK’s testing service in these months. Figure 3a shows that when only 40% of these cases request tests, capacity is sufficient for the entire year. However, as this proportion increases, capacity becomes insufficient for predicted demand. When 60% request tests, demand in December 2020 and January 2021 exceeds capacity by 58,308 (95%CI 3,362 – 127,505) and 9,121 (95%CI -39,541 – 71,798) tests per day ( Figure 3b). For 80% ( Figure 3c), the daily average demand for tests exceeds capacity in five consecutive months (October 2020 to February 2021), with a peak of 147,240 (95%CI 73,978 – 239,502) tests per day above capacity expected in December 2020. If all individuals experiencing non-COVID-19 cough or fever request a test, we estimate that there will only be a significant capacity surplus in the summer months of July and August 2020 and June 2021.

### Total UK testing demand including symptomatic COVID-19 cases

**Figure 4.**
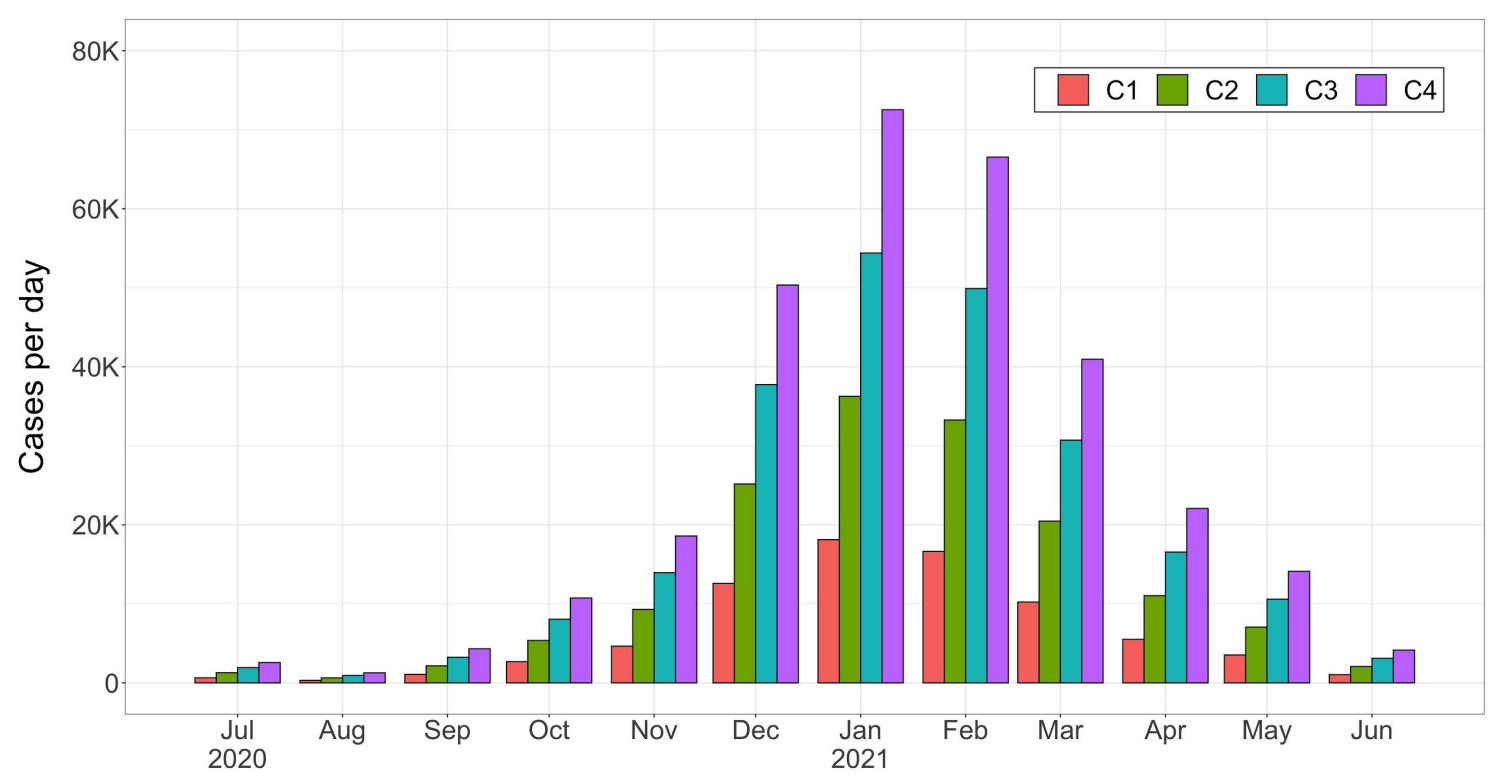
Four scenarios (C1 to C4) for average daily COVID-19 incidence in the UK shown for each month.

The four scenarios (C1-C4) for a winter COVID-19 epidemic in the UK which were explored in this study are shown in Figure 4. All scenarios follow the same epidemic curve shape with the highest average daily incidences between December 2020 and March 2021 and peak incidences in January of 18,134 (in scenario C1), 36,268 (C2), 54,402 (C3) and 72,536 (C4) cases per day.

**Figure 5.**
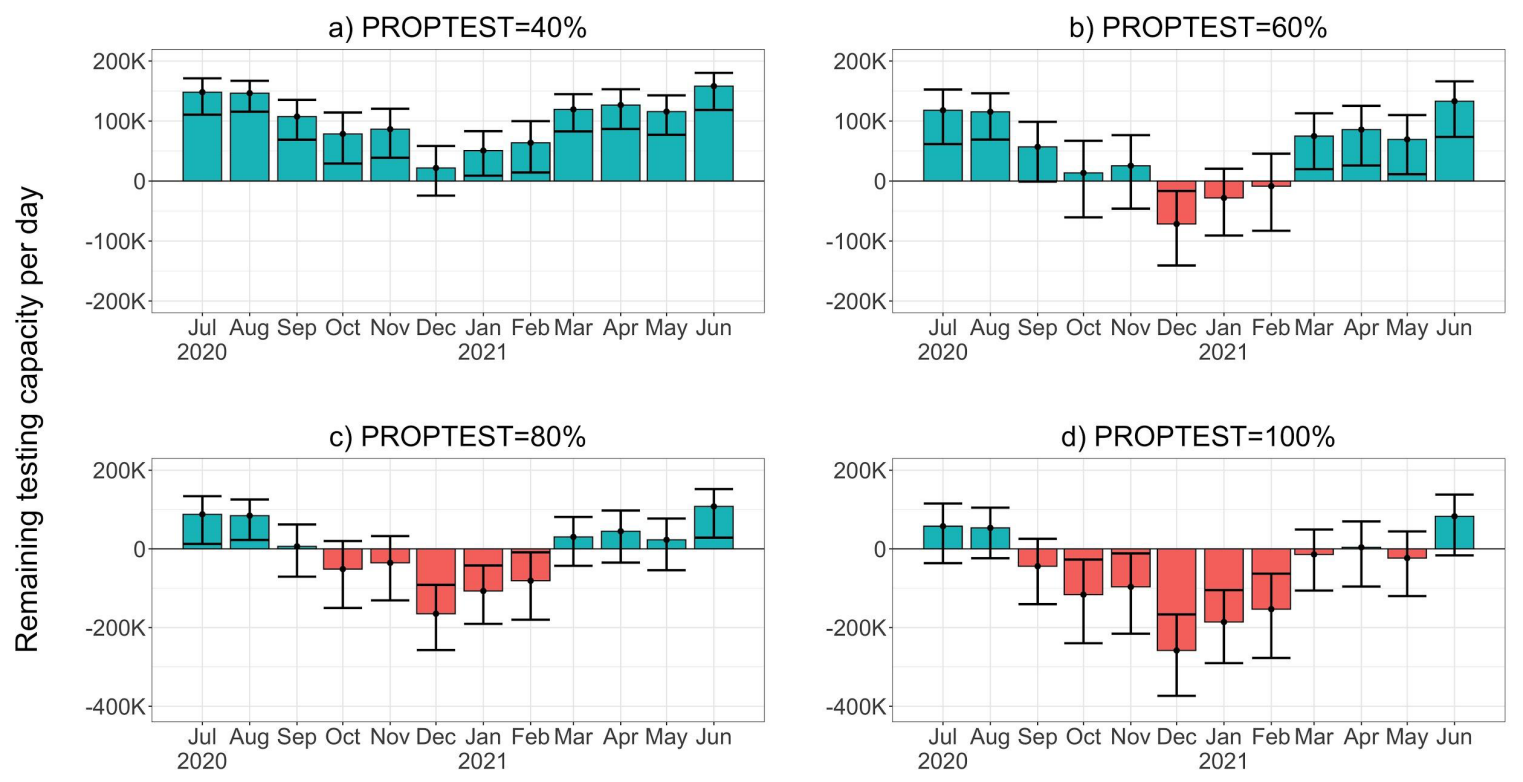
Remaining UK Pillar 1 and 2 testing capacity (thousands of tests) per day after testing baseline (non-COVID-19) cases of cough or fever and symptomatic COVID-19 cases in the UK each day for scenario C2. Panels **a**) to **d**) show results for four values of the proportion of cases requesting tests (PROPTEST). Blue and red bars indicate that testing demand is within or in excess of capacity in August 2020, respectively. Note that panel **a**) has a different y-axis scale to panels **b**) - **d**).

For the midrange C2 scenario, the remaining testing capacity available after testing baseline cough or fever cases and symptomatic COVID-19 cases is shown in Figure 5 (results for all other scenarios were similar and are included in *Extended data,* S4 ^9^). These results were similar to those for baseline cough or fever cases only ( Figure 3). Capacity is not predicted to be exceeded when only 40% of cough or fever cases and symptomatic COVID-19 cases request tests. When 60% of cases request tests, there is a predicted daily demand in December 2020 of 71,522 (95%CI 16,576 – 140,719) tests above capacity and the additional COVID-19 demand pushes total demand in February 2021 above capacity. When 80% of cases request tests, we see an increased deficit during the epidemic’s peak months of December to January and, when 100% request a test, UK testing capacity is predicted to be severely strained from September 2020 to May 2021.

**Figure 6.**
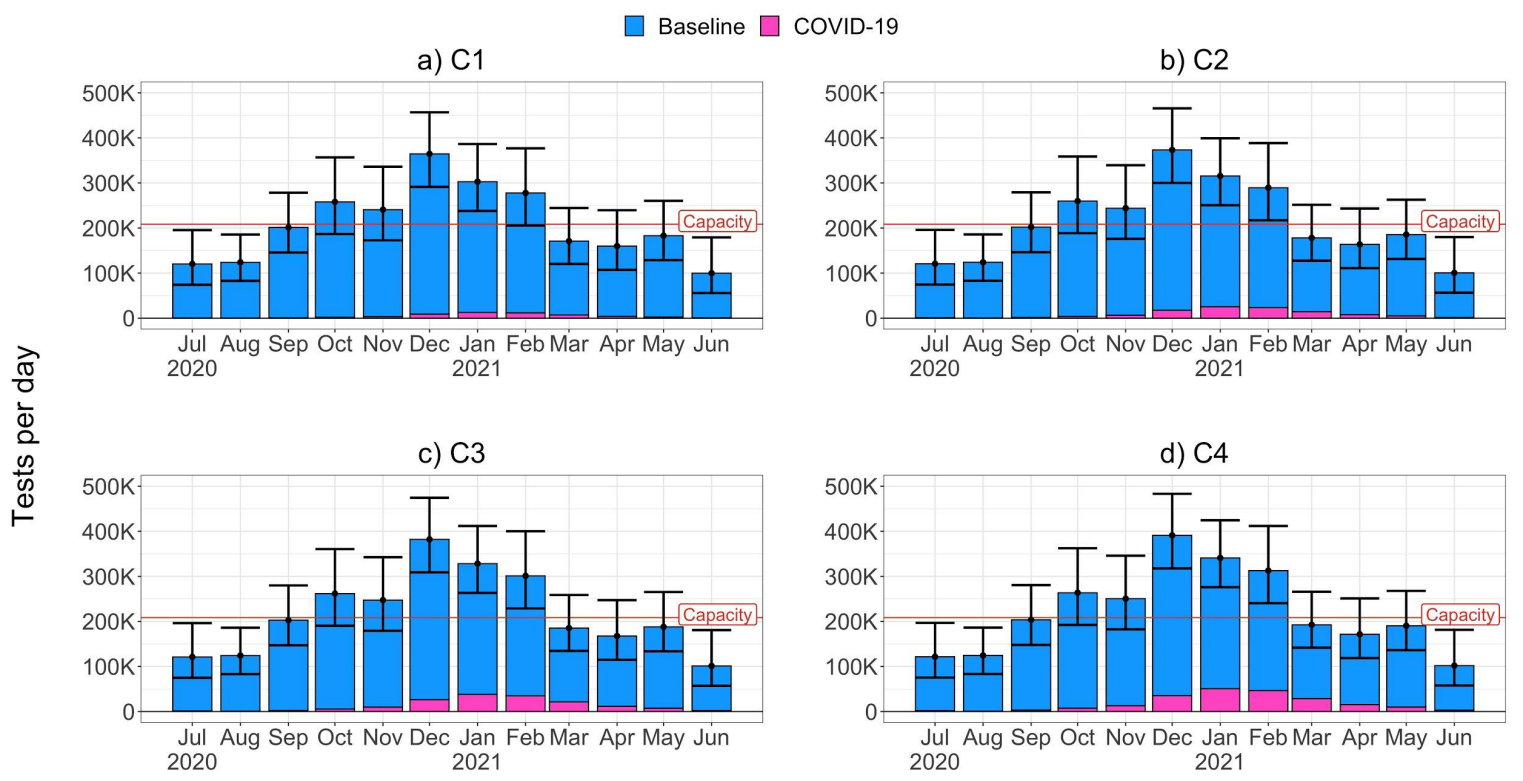
Predicted daily testing demand (thousands of tests) due to baseline cough or fever cases (blue) and symptomatic COVID-19 cases (pink) in the UK, assuming that 80% of cases request tests. Panels **a**) to **d**) show results for scenarios C1 to C4. Daily UK Pillar 1 and 2 testing capacity estimates from August 2020 are marked with a red line.

The relatively small contribution of symptomatic COVID-19 cases to total predicted testing demand in the UK for scenario C2 was also found for all other COVID-19 transmission scenarios, shown in Figure 6, for an assumed value of 80% of cases requesting a test. The more severe C3 and C4 scenarios result in higher total demand in January 2021 of 328,227 (95%CI 263,345 – 411,797) and 340,921 (95%CI 276,039 – 424,491) tests per day, respectively, compared to 302,839 (95%CI 237,957 – 386,409) in the C1 scenario. While these increases are not negligible, the total testing demand is predominantly driven by baseline cough or fever cases, and the overall effect of increased COVID-19 transmission between these scenarios is small. For example, in the highest transmission scenario, C4, and peak COVID-19 incidence in January 2021, only 14.9% of tests are expected to be requested by COVID-19 cases. This is a result of the high incidence of baseline cough or fever cases during the second wave peak months of December 2020 to February 2021. Consequently, relative to UK testing capacity in August 2020, the overall picture remains the same across the next year for all four scenarios, with the period October to February at high risk of surpassing UK testing capacity. As the proportion of cases requesting tests is applied to both baseline and symptomatic COVID-19 cases, symptomatic COVID-19 cases contribute the same proportion of the total testing demand for all other values of the proportions of cases requesting tests, although total demand varies significantly with this proportion (see *Extended data,* S5 ^9^).

### Total UK testing demand in a low baseline cough or fever incidence scenario

**Figure 7.**
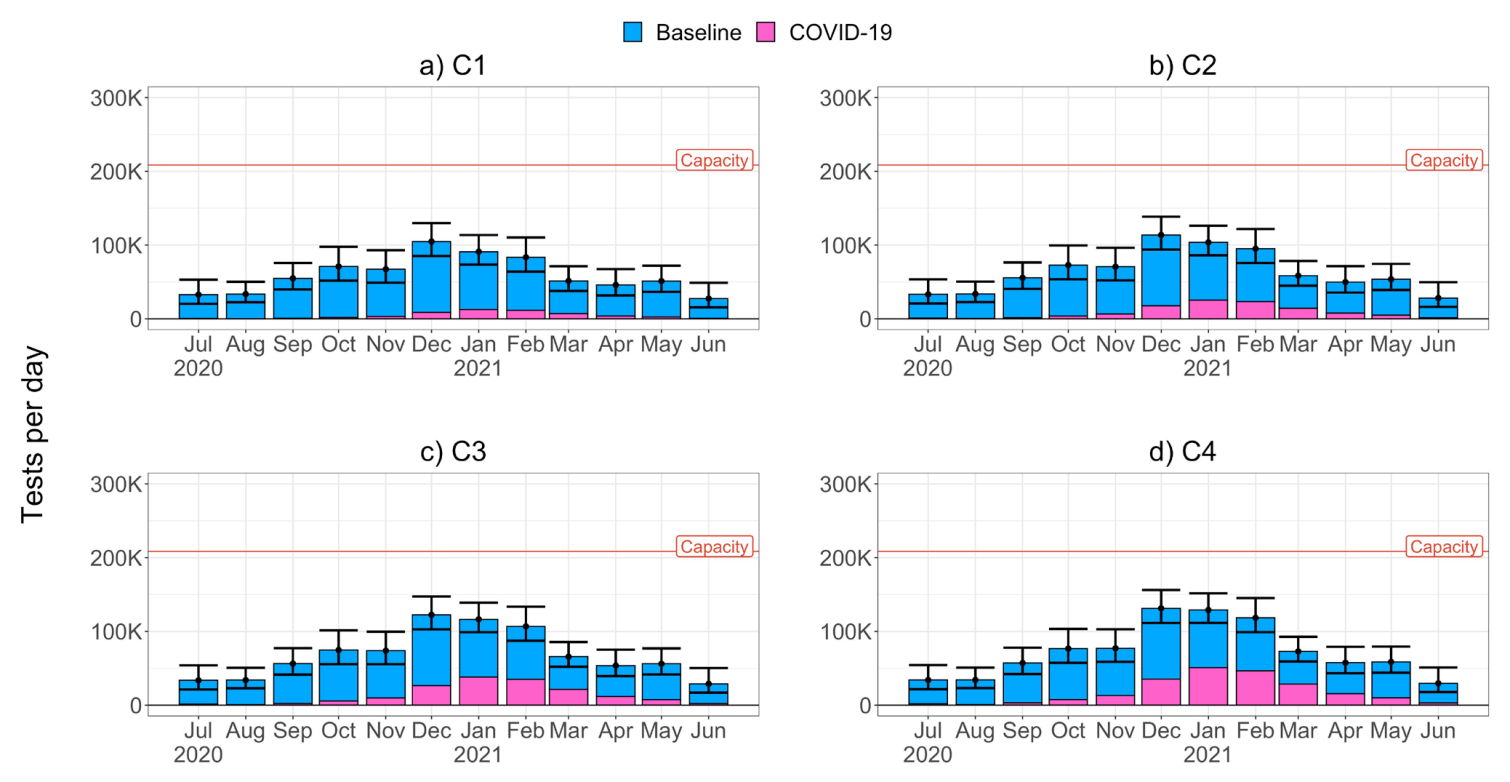
Predicted daily testing demand (thousands of tests) due to baseline cough or fever cases (blue) and symptomatic COVID-19 cases (pink) in a low baseline cough or fever incidence scenario (73% lower than 2018–2019 incidence) in the UK, assuming that 80% of cases request tests.

The low baseline cough or fever incidence scenario which explored the effect of a 73% reduction in cough or fever incidence relative to the historical baseline is shown in Figure 7. For a C4 transmission scenario with 80% of cases requesting tests, the estimated total UK testing demand was below capacity reaching 129,115 (95%CI 111,596 - 151,679) tests per day in January 2021. This lower cough or fever incidence scenario results in significantly lower levels of total testing demand when compared to the high scenario estimates ( Figure 6) which were based on the historical baseline cough or fever incidence. These lower estimated incidences of cough or fever would result in COVID-19 cases playing a more significant role in driving testing demand, with COVID-19 cases constituting 26%, 39%, and 39% of tests requested in December, January and February, respectively.

## Discussion

In this study, we estimate the baseline incidence of cough or fever cases and their potential impact on COVID-19 diagnostic testing services in the UK in July 2020 – June 2021. Our results show that if the baseline incidence of cough and fever between September 2020 and February 2021 is similar to incidences in previous years, it may place a significant strain on UK testing capacity. Under these conditions we estimate that if more than 80% of people with symptoms request a test, daily demand for COVID-19 swab tests will exceed the testing capacity available in August 2020 by a significant margin for five months. However, if the baseline incidence of cough or fever consistently remains at significantly lower levels than in previous years then capacity is likely to be sufficient. We find that testing demand in both baseline incidence scenarios will be predominantly driven by baseline cough or fever incidence, rather than symptomatic COVID-19 cases, and that the proportion of people with symptoms who request a test is a key determinant of demand. To our knowledge, this is the first paper published quantifying baseline cough or fever cases in the UK and their impact on COVID-19 diagnostic testing services.

The strong seasonal trend in baseline incidence of cough or fever cases in the UK is a clear indicator of the challenges which the UK’s testing services will face in the autumn and winter months of 2020–2021. Our results show that while the UK’s diagnostic testing capacity in August 2020 may be sufficient for July to September 2020, this is may not be the case during the winter period. If capacity is exceeded to the extent predicted in our results, a large backlog of unprocessed tests can be anticipated and a significant proportion of COVID-19 positive cases are likely to remain untested. Prompt identification of cases is critical for effective contact tracing and real-time visualisation of epidemiological trends to inform national and local-level public health interventions. It is therefore imperative that the UK’s testing capacity continues to be scaled up to ensure that there is sufficient capacity to respond to this predicted rise in testing demand and ensure that the detection of COVID-19 cases is not compromised. Delays in testing, due to lack of capacity, will negatively affect the performance of the track and trace system, may also disincentivise people from getting tested and may result in unnecessarily extended self-isolation of COVID-19 negative households. The UK testing strategy has acknowledged the need to expand testing capacity rapidly ^18^ and appears to be on track to reach a PCR testing capacity of half a million tests per day by the end of 2020 ^19^. Our calculations show that if this additional capacity is achieved, it would significantly reduce the risk of testing backlogs over the winter period. However, under high levels of COVID-19 transmission and a baseline incidence of cough or fever which approaches historical levels, it is unlikely to be sufficient to also cover routine testing of asymptomatic health and care professionals ^20^. Clearly, any improvements in the UK’s testing system must also be matched by the creation of an effective contact tracing system for identifying clusters at the local level and preventing onwards transmission, and by providing support to people to self-isolate – testing alone will not control transmission in the UK.

Our results show that testing demand is likely to be driven by the proportion of people with symptoms that request a test. An effective public health response to COVID-19 in the UK requires all individuals experiencing symptoms to request a test promptly after symptoms begin and our estimates for higher values of the proportion requesting tests should be considered as a necessary requirement for the UK’s testing capacity. Therefore, in addition to scaling up capacity, the UK’s response must also ensure that a high proportion of symptomatic people are requesting tests to begin with. Estimates for this proportion are not widely documented, but one recent study found that “that only about 40% of those who report classic COVID-19 symptoms go on to receive a test” ^21^. This low value is concerning for COVID-19 control and would suggest that the results in our study for scenarios with lower values of the proportion of cough or fever cases that request a test may be more accurate, and consequently that demand for testing would lie within capacity. However, understanding this test-seeking behaviour is clearly important and should be studied further through future weekly follow-up community studies structured similarly to the Bug Watch study. The potentially high baseline incidence of cough or fever cases reported in our analysis highlights the scale of this issue, and culturally and linguistically appropriate public engagement campaigns, as well as accessible and rapid test-ordering systems, will be critical to a successful response.

In this study, we explore a scenario in which the baseline incidence of cough or fever during the study period of 2018–19 is representative of 2020–2021 and a second scenario in which the incidence during 2020–2021 is 73% lower. Which of these scenarios is most representative of demand in the winter 2020–2021 is likely to depend on the type of public health restrictions which are implemented and how well they are observed by the UK population. The implementation of public health interventions in the UK during autumn 2020 varied geographically with an easing of restrictions in most of the UK during July–September 2020 followed by further restrictions in some higher transmission areas in November and December 2020. Many measures, such as social distancing and bans on mass gatherings, will continue regardless of local restrictions. These public health interventions and changes in behaviour have been impacting upon the incidence of other respiratory pathogens globally, with reduced influenza activity reported in the United States, Australia, Chile and South Africa between June-August 2020 ^22^ and UK surveillance data from 2019 to 2020 suggesting low levels of influenza activity in the community ^23^. This is consistent with the preliminary results from the Virus Watch cohort study which were used in the low incidence scenario. The lowest reduction of 34% was in September 2020 which may be explained by the fact that children and young adults can drive transmission of influenza and other seasonal respiratory infections. The reopening of schools and universities in the UK in September 2020 may therefore have maintained baseline cough or fever incidence in this month closer to 2018–19 levels. It is consequently likely that the future incidence of baseline cough or fever cases with an infectious aetiology will depend on the extent to which schools and universities are kept open. Our analysis provides estimates of predicted total testing demand for the reasonable best and worst case of baseline cough and fever incidence and clearly shows that the strain on testing capacity is highly dependent on the extent to which these other respiratory pathogens continue to be transmitted.

The COVID-19 transmission scenarios explored in this study are speculative and reflect uncertainty about the potential size and timing of a second COVID-19 wave. However, they present a range of reasonable scenarios based on previous modelling predictions ^14^ that are consistent with the expectation that a second wave will have a lower peak incidence and flatter epidemic curve than the first wave in March – July 2020 in the UK ^24^. Our results show that total testing demand is relatively insensitive to COVID-19 transmission and that, even in more severe scenarios, testing demand due to baseline cough or fever cases will outweigh demand due to symptomatic COVID-19 cases.

A limitation of our study was that participants were more likely to be older, female, healthier and living in less deprived areas than the general population of England. To account for age and sex, we adjusted for these variables in our incidence estimates. There were also a disproportionately high number of white participants included in the study – meaning that ethnic minority communities that are known to have been particularly adversely affected by COVID-19 were underrepresented ^25^. Consequently, our results do not account for possible differences in the incidence of baseline cough and fever in these groups. Another possible limitation of this study is that a cough was defined as ‘incident’ rather than ‘continuous’ (lasting more than one hour or three or more coughing episodes in 24 hours), therefore potentially differing from the UK’s COVID-19 diagnostic symptomatology. We may consequently overestimate the number of cough cases who would require a test under current NHS guidance. We also note that our estimates of testing demand were largely driven by cough symptoms, as fever was comparatively less common. Data was not collected on altered or lost sense of smell or taste, but we expect this to be rare in comparison to symptoms of cough or fever.

In conclusion, our study provides estimates of the baseline incidence of cough or fever in the general population in the UK. Our estimates indicate that, if baseline cough or fever incidence is maintained at 2018–2019 levels, the UK’s COVID-19 testing capacity in August 2020 is insufficient for high predicted demand in winter 2020–2021 . However, if cough or fever incidence is maintained at the lower levels observed in October and November 2020 then it is likely to be sufficient, even in the most severe COVID-19 transmission scenario explored in this analysis. This study highlights the need to ensure that a high proportion of people with symptoms request tests and that sufficient testing capacity must be available for testing baseline cough or fever cases. Otherwise, compounded by high COVID-19 levels projected in a second wave, UK testing capacity could be overwhelmed leading to failure of the NHS Test and Trace service and an inability to control the further spread of COVID-19.

### Patient and public involvement

Participants were not directly involved in design of this study although feedback was collected at two time points during follow-up. Please see the Bug Watch community cohort study protocol for more information ^7^.

## Data availability

### Underlying data

Open Science Framework: Impact of baseline cases of cough and fever on UK COVID-19 diagnostic testing rates: estimates from the Bug Watch community cohort study - Supplementary material, code and data. https://doi.org/10.17605/OSF.IO/5J6DY ^9^

The repository contains the following underlying data:

- data1_surv_com_week.csv (Survey completion rates)
- data2_bl_exp.csv (Anonymised individual characteristics of participants)
- data3_daily.csv (Daily symptom reports)
- ONS_England_mid-2019_extracted_sex-reg-age.csv (Demographic data extracted from ONS mid-2019 estimates for England, Source: Office for National Statistics licensed under the Open Government Licence v.3.0)
- ONS_UK_mid-2019_extracted_sex-age.csv (Demographic data extracted from ONS mid-2019 estimates for the United Kingdom, Source: Office for National Statistics licensed under the Open Government Licence v.3.0)
- Virus_watch_results_adj_month_2020-12-22.csv (Virus Watch cohort study estimates for the incidence of cough or fever cases in 2020).
- VW_monthly_cough_prop.csv (Estimates of the proportion of cough or fever cases which are not COVID-19 cases from Virus Watch cohort study).

### Extended data

Open Science Framework: Impact of baseline cases of cough and fever on UK COVID-19 diagnostic testing rates: estimates from the Bug Watch community cohort study - Supplementary material, code and data. https://doi.org/10.17605/OSF.IO/5J6DY ^9^

This repository contains the R script used to conduct this analysis, supplementary material, underlying data and STROBE cohort study reporting checklist. 

This project contains the following extended data:

- S1. Study population information
- S2. Age-specific incidence rates of cough or fever
- S3. COVID-19 incidences for scenarios C1 to C4
- S4. Full exploration of proportion requesting test values for each scenario: C1, C3 and C4
- S5. Full exploration of scenarios C1, C3 and C4 for each proportion requesting test value: 40%, 60%, 100%
- S6. Estimated cough incidence rates in England
- S7. Estimated fever incidence in England

Data are available under the terms of the Creative Commons Zero "No rights reserved" data waiver (CC0 1.0 Public domain dedication).

## References

[1] NHS Online: Check if you or your child has coronavirus (COVID-19) symptoms - NHS. (accessed on 16 August 2020). Reference Source

[2] NHS Online. Get a free NHS test today to check if you have coronavirus (COVID-19) - NHS. (accessed on 3 September 2020). Reference Source

[3] NHS Online. When to self-isolate and what to do - Coronavirus (COVID-19) - NHS. (accessed on 3 September 2020). Reference Source

[4] NHS England and NHS Improvement: Guidance and standard operating procedure - COVID-19 virus testing in NHS laboratories (Version 1.0 VIRUS TESTING 16 March 2020).2020; 001559. Reference Source

[5] UK Government: Foreign Secretary’s statement on coronavirus (COVID-19): 16 April 2020 - GOV.UK.2020; (accessed on 17 August 2020). Reference Source

[6] Ma X Conrad T Alchikh M: Can we distinguish respiratory viral infections based on clinical features? A prospective pediatric cohort compared to systematic literature review. *Rev Med Virol.* 2018;28(5):e1997. 10.1002/rmv.1997 30043515 PMC7169127

[7] Smith CM Conolly A Fuller C: Symptom reporting, healthcare-seeking behaviour and antibiotic use for common infections: protocol for Bug Watch, a prospective community cohort study. *BMJ Open.* 2019;9(5):e028676. 10.1136/bmjopen-2018-028676 31123004 PMC6537990

[8] Smith CM: Unpublished. Incidence, healthcare-seeking behaviours, antibiotic use and natural history of common infection syndromes in England: Results from the Bug Watch community cohort study. 10.1186/s12879-021-05811-7 PMC7820521 33482752

[9] Eyre M Burns R Kirkby V: Impact of baseline cases of cough and fever on UK COVID-19 diagnostic testing rates: estimates from the Bug Watch community cohort study - Supplementary material, code and data.2020 10.17605/OSF.IO/5J6DY PMC7890379 33655079

[10] Public Health England: Surveillance of influenza and other respiratory viruses in the UK Winter 2018 to 2019.2019 Reference Source

[11] Office for National Statistics: Population estimates for the UK, England and Wales, Scotland and Northern Ireland. In press; (accessed on 10 August 2020). Reference Source

[12] Lumley T: Package ‘survey’: Analysis of Complex Survey Sample.2019.

[13] UK Government: Coronavirus cases in the UK: daily updated statistics - GOV.UK.2020; (accessed on 17 August 2020). Reference Source

[14] Academy of Medical Science: Preparing for a challenging winter 2020/21.2020;79 Reference Source

[15] Menni C Sudre CH Steves CJ: Quantifying additional COVID-19 symptoms will save lives. *Lancet.* 2020;395(10241):e107–e108. 10.1016/S0140-6736(20)31281-2 32505221 PMC7272184

[16] Team RC: R: A language and environment for statistical computing.2017.

[17] Wickham H Averick M Bryan J: Welcome to the {tidyverse}. *J Open Source Softw.* 2019;4:1686 10.21105/joss.01686

[18] HM Government: Our Plan to Rebuild: The UK Government’s COVID-19 recovery strategy.2020 Reference Source

[19] UK Government: Coronavirus (COVID-19) in the UK. (accessed on 22 December 2020). Reference Source

[20] NHS England: Third Phase of NHS Response to COVID19.2020;1–13. Reference Source

[21] Varsavsky T Graham MS Canas LS: Detecting COVID-19 infection hotspots in England using large-scale self-reported data from a mobile application: a prospective, observational study. *Lancet Public Health.* 2020;6(1):e21–e29. 10.1016/S2468-2667(20)30269-3 33278917 PMC7785969

[22] Olsen SJ Azziz-Baumgartner E Budd AP: Decreased Influenza Activity During the COVID-19 Pandemic — United States, Australia, Chile, and South Africa, 2020. *MMWR Morb Mortal Wkly Rep.* 2020;69(37):1305–1309. 10.15585/mmwr.mm6937a6 32941415 PMC7498167

[23] Public Health England: Surveillance of influenza and other respiratory viruses in the United Kingdom: Winter 2019 to 2020.2020 Reference Source

[24] Jit M Jombart T Nightingale ES: Estimating number of cases and spread of coronavirus disease (COVID-19) using critical care admissions, United Kingdom, February to March 2020. *Euro Surveill.* 2020;25(18):2000632. 10.2807/1560-7917.ES.2020.25.18.2000632 32400358 PMC7219029

[25] Public Health England: Beyond the data: Understanding the impact of COVID-19 on BAME groups About Public Health England.2020;69 Reference Source

