# Supplementary material for: Impact of baseline cases of cough and fever on UK COVID-19 diagnostic testing rates: estimates from the Bug Watch community cohort study
Source: Wellcome Open Res. 2021 Jan 25;5:225. Originally published 2020 Sep 24. [Version 2] doi: 10.12688/wellcomeopenres.16304.2 (PMC7890379; doi:10.12688/wellcomeopenres.16304.2)
Supplement: Peer review history

# Peer review: Reviewer response for version 2

**DOI:** 10.21956/wellcomeopenres.18263.r42288

**Published:** 2021-02-17

**Authors:** Cheryl Cohen

**Affiliations:** 1 Centre for Respiratory Diseases and Meningitis, National Institute for Communicable Diseases of the National Health Laboratory Service, Johannesburg, South Africa

I have reviewed the authors responses and I am happy with them.

Is the work clearly and accurately presented and does it cite the current literature?

Yes

If applicable, is the statistical analysis and its interpretation appropriate?

I cannot comment. A qualified statistician is required.

Are all the source data underlying the results available to ensure full reproducibility?

Yes

Is the study design appropriate and is the work technically sound?

Partly

Are the conclusions drawn adequately supported by the results?

Partly

Are sufficient details of methods and analysis provided to allow replication by others?

Yes

Reviewer Expertise:

Infectious disease epidemiology, viral respiratory illness.

I confirm that I have read this submission and believe that I have an appropriate level of expertise to confirm that it is of an acceptable scientific standard.

# Peer review: Reviewer response for version 1

**DOI:** 10.21956/wellcomeopenres.17916.r40603

**Published:** 2020-10-19

**Authors:** Angela Raffle

**Affiliations:** 1 Consultant in Public Health, The NHS Screening Programmes, University of Bristol Medical School Population Health Sciences, Bristol, UK

This article is well summarised in the abstract, and in the text provided by Reviewer 1. 

**General comment:**

- This is a clear and well-written paper that addresses an important issue. I agree with the points made by Reviewer 1.

 

**Specific comments:**

 Abstract:

- If possible within the word count, it would be good to include that the pillars 1 plus 2 capacity estimate being used is 1.4 million tests/week. 

 

 Introduction:

- ‘The NHS Test and Trace service will trace and notify close recent contacts of anyone who tests positive for coronavirus to self-isolate for 14 days.’ It would be more correct to say that they ‘aim to’ rather than they ‘will’. Also, adding the words ‘instructing them’ to self-isolate would be less cryptic. 
- Last paragraph, would it not be more correct to say ‘Bug Watch allows us to estimate’  (rather than quantify), as said later. The sample was 873 participants, with potential for selection bias, so extrapolating to the entire population and to different years gives only an estimate.  

 

 Methods: 

- There should be mention in the methods or results sections of whether, and potentially how, selection bias may have skewed the measured incidence rates in Big Watch. This is touched on in the discussion, but it would be better to state it earlier in the paper. 
- Baseline incidence of cough or fever in England -  I found this paragraph hard to follow. I could not quite grasp how the term ‘follow-up’ was being used. Is this about excluding days from the denominator? 
- Also, was cough or fever defined as any mention of these symptoms i.e. one day of reporting only? This needs to be specified in the methods section because this could be quite a ‘low bar’ for defining cough or fever. There is mention in the discussion, but I found it a bit ambiguous – was the ‘continuous’ cough definition used? This needs to be clear in the methods section. 
- Also, mention of whether 2018/19 a high, low, or medium year for respiratory illnesses in general, would be helpful. 
- Is it possible to use general practice morbidity data to add further insight into the relationship between the incidence of new cough or fever from the Bug Watch study, compared with GP consultation rates? This could give some insight into whether the 40, 60, or 80 percent seeking testing is most likely to be accurate. This is not an essential change, but would be a 'nice to have'.
- Covid 19 estimates – I am not qualified to comment on the validity of these estimates

Discussion:

- At one point the term ‘antigen’ testing is used – does this mean PCR testing for viral RNA? It is potentially confusing to refer to this as antigen testing. 
- The discussion only considers the need for greater testing capacity. It would be worth also mentioning the need to use tests to best effect. For example, as data develops on the predictive value of certain symptoms for ruling in and ruling out SARS-CoV-2 there may be scope for refining who should be offered a test e.g. cough with phlegm was presumably included in Bug Watch but is a dry cough more indicative of SARS-C0V-2? Also the timing of the test matters – if there is a delay in seeking a test then the result would not shorten the isolation period and it is too late to effectively act on all the contacts etc. Also, there has been considerable criticism of Test and Trace, so perhaps a phrase emphasising that a test alone is pointless. Testing must form part of a well-coordinated system that reliably leads to effective contact tracing, to prompt local action for dealing with clusters, and to support for those who are needing to self-isolate.  

Is the work clearly and accurately presented and does it cite the current literature?

Yes

If applicable, is the statistical analysis and its interpretation appropriate?

Partly

Are all the source data underlying the results available to ensure full reproducibility?

Yes

Is the study design appropriate and is the work technically sound?

Yes

Are the conclusions drawn adequately supported by the results?

Partly

Are sufficient details of methods and analysis provided to allow replication by others?

Yes

Reviewer Expertise:

Public Health Screening Programmes, NHS Public Health service provision including general (non-specialist) communicable disease control.

I confirm that I have read this submission and believe that I have an appropriate level of expertise to confirm that it is of an acceptable scientific standard.

# Response

**Published:** 2021-01-15

**Authors:** Max Eure

**Affiliations:** Lancaster University, UK

Many thanks for the time you have spent reviewing our paper and your very helpful and constructive comments on the paper. We have tried to address all of the points you have raised and to update the paper as much as is reasonably possible within the rapidly changing context. There is now an additional analysis which explores a lower (73% lower) cough or fever incidence scenario based on preliminary estimates from the Virus Watch cohort study we are running at UCL. We provide numbered point by point responses to your comments below.

**Reviewer comment 1:**

 Abstract - If possible within the word count, it would be good to include that the pillars 1 plus 2 capacity estimate being used is 1.4 million tests/week. 

**Author response 1:**

 This has been added. We have also made clear that this was the capacity in August 2020.

****

**Reviewer comment 2:**

 Introduction - ‘The NHS Test and Trace service will trace and notify close recent contacts of anyone who tests positive for coronavirus to self-isolate for 14 days.’ It would be more correct to say that they ‘aim to’ rather than they ‘will’. Also, adding the words ‘instructing them’ to self-isolate would be less cryptic. Last paragraph, would it not be more correct to say ‘Bug Watch allows us to estimate’  (rather than quantify), as said later. The sample was 873 participants, with potential for selection bias, so extrapolating to the entire population and to different years gives only an estimate.

**Author response 2:**

 Thank you for these suggestions, we have added both of these corrections into the introduction.

**Reviewer comment 3:**

 Methods - There should be mention in the methods or results sections of whether, and potentially how, selection bias may have skewed the measured incidence rates in Big Watch. This is touched on in the discussion, but it would be better to state it earlier in the paper.

**Author response 3:**

 Thank you for this comment, we have highlighted the potential for selection bias and commented on its potential skew in the methods section.

****

**Reviewer comment 4:**

 Methods - Baseline incidence of cough or fever in England -  I found this paragraph hard to follow. I could not quite grasp how the term ‘follow-up’ was being used. Is this about excluding days from the denominator?

**Author response 4:**

 It should hopefully be clearer now that we excluded the first ten days for all participants after they entered the study - “The first ten days of follow-up after each participant was recruited into the study were excluded to remove prevalent infection syndromes.”

****

**Reviewer comment 5:**

 Methods - Also, was cough or fever defined as any mention of these symptoms i.e. one day of reporting only? This needs to be specified in the methods section because this could be quite a ‘low bar’ for defining cough or fever. There is mention in the discussion, but I found it a bit ambiguous – was the ‘continuous’ cough definition used? This needs to be clear in the methods section.

**Author response 5:**

 Thank you for pointing this out - we have clarified that a single day of reporting the symptom counts as a case of cough or fever. The symptom definition used for cough is described in the first paragraph of the methods section.

**Reviewer comment 6:**

 Methods - Also, mention of whether 2018/19 a high, low, or medium year for respiratory illnesses in general, would be helpful.

**Author response 6:**

 Thank you for this helpful suggestion. Please see the sentence we have added in the methods section: “Public Health England reported that there were “low to moderate levels of influenza activity” in the 2018-2019 influenza season, which was comparable to the 2017-2018 season and higher than all other seasons since 2010-2011”.

**Reviewer comment 7:**

 Methods - Is it possible to use general practice morbidity data to add further insight into the relationship between the incidence of new cough or fever from the Bug Watch study, compared with GP consultation rates? This could give some insight into whether the 40, 60, or 80 percent seeking testing is most likely to be accurate. This is not an essential change, but would be a 'nice to have'.

**Author response 7:**

 We have added a new reference in the discussion section which estimates  “that only about 40% of those who report classic COVID-19 symptoms go on to receive a test”. We then comment on the implications of this on COVID-19 control and our results, and stress the importance of future studies looking at test-seeking behaviour and comment on how they can be structured.

**Reviewer comment 8:**

 Discussion - At one point the term ‘antigen’ testing is used – does this mean PCR testing for viral RNA? It is potentially confusing to refer to this as antigen testing. 

**Author response 8:**

 This has been corrected.

****

**Reviewer comment 9:**

 Discussion - The discussion only considers the need for greater testing capacity. It would be worth also mentioning the need to use tests to best effect. For example, as data develops on the predictive value of certain symptoms for ruling in and ruling out SARS-CoV-2 there may be scope for refining who should be offered a test e.g. cough with phlegm was presumably included in Bug Watch but is a dry cough more indicative of SARS-C0V-2? Also the timing of the test matters – if there is a delay in seeking a test then the result would not shorten the isolation period and it is too late to effectively act on all the contacts etc. Also, there has been considerable criticism of Test and Trace, so perhaps a phrase emphasising that a test alone is pointless. Testing must form part of a well-coordinated system that reliably leads to effective contact tracing, to prompt local action for dealing with clusters, and to support for those who are needing to self-isolate.  

**Author response 9:**

 We have addressed this important point by inserting some additional text in the discussion which highlights that testing is only useful when accompanied by an effective contact tracing and a system which supports people to self-isolate.

# Peer review: Reviewer response for version 1

**DOI:** 10.21956/wellcomeopenres.17916.r40629

**Published:** 2020-10-12

**Authors:** Cheryl Cohen

**Affiliations:** 1 Centre for Respiratory Diseases and Meningitis, National Institute for Communicable Diseases of the National Health Laboratory Service, Johannesburg, South Africa

**Impact of baseline cases of cough and fever on UK COVID-19 diagnostic testing rates: estimates from the Bug Watch community cohort study**

 This paper uses data from a community cohort study to estimate the number of baseline cough and fever cases expected to occur in the 2020-2021 winter in the UK in order to infer the minimum COVID-19 testing capacity needed during the winter months. This is an important question and the approach taken is generally scientifically robust. The paper is nicely written and the approach is generally sound. I do have some suggestions below.

Major comments

 The authors assume that the baseline rates of fever and cough in the upcoming winter will be similar to those of previous years with additional COVID-19-related burden superimposed. Data from the Southern Hemisphere 2020 winter however suggest that this may not be the case. Most Southern Hemisphere countries did not experience an influenza season in the 2020 winter and in many countries levels of ILI consultations were at all time low levels ^1^. The authors should expand the abstract, introduction and discussion to consider this possibility further. It does not invalidate the relevance of the analysis but it should be clear that the projects presented may not be a true minimum estimate as it is possible that while COVID circulates other respiratory viruses may be at all-time low levels as may cases of fever and cough. The reason for the Southern Hemisphere observations are not known and it is also unclear what level of interventions will be in place to control respiratory virus transmission during the upcoming UK winter season. Hence it is unclear whether the UK will see a reduction in respiratory illness. The authors should reposition the paper to include this possibility in introduction discussion and abstract.

 This should also be included as a scenario in the analysis – i.e. baseline fever and cough is 50% OR 20% of historical numbers.

 Methods: ***UK testing demand due to baseline cough and fever cases*** – the authors explore a range of scenarios for the proportion of cough and fever seeking testing. I think the explored ranges are reasonable but can the authors provide some context please (perhaps from other studies) on proportion of fever and cough cases which usually seek medical care (and perhaps get a sample for testing) in UK and any estimates (if they exist) or thoughts of how health seeking could increase (or decrease) in response to COVID to help contextualise which of these estimates they think more likely.

 Could you use your study data from the summer period to look at the testing demand in the months which have already past? I.e. compare the rates for months which have past (March-August) from your study to the actual demand for testing experienced to date in the UK to see how the numbers compare and inform our thinking about how they might compare going forward. I.e. have the numbers to date been more or less than you would have predicted from your data?

Minor comments

 Discussion line 3 – suggest change “will place a strain” to may place a strain.

 Discussion – perhaps suggest some ways that UK could collect data on proportion of people with fever and cough who do get a test (e.g. through online platforms like your study) as these data may be useful to inform the public health response.

 Discussion paragraph 5: “Our results show that total testing demand is relatively insensitive to COVID-19 transmission” this is an interesting result – are there any published studies which have found this – perhaps again from the Southern Hemisphere – I agree this is what would be expected but it would interesting to see if this happened anywhere. COVID detection rates in different settings could be helpful but would need to know the case definitions used for testing and health seeking behaviour which data are generally not available.

Is the work clearly and accurately presented and does it cite the current literature?

Yes

If applicable, is the statistical analysis and its interpretation appropriate?

I cannot comment. A qualified statistician is required.

Are all the source data underlying the results available to ensure full reproducibility?

Yes

Is the study design appropriate and is the work technically sound?

Partly

Are the conclusions drawn adequately supported by the results?

Partly

Are sufficient details of methods and analysis provided to allow replication by others?

Yes

Reviewer Expertise:

Infectious disease epidemiology, viral respiratory illness.

I confirm that I have read this submission and believe that I have an appropriate level of expertise to confirm that it is of an acceptable scientific standard, however I have significant reservations, as outlined above.

## References

[1] : Decreased Influenza Activity During the COVID-19 Pandemic - United States, Australia, Chile, and South Africa, 2020. *MMWR Morb Mortal Wkly Rep*.2020;69(37) : 10.15585/mmwr.mm6937a6 1305-1309 10.15585/mmwr.mm6937a6 32941415PMC7498167

# Response

**Published:** 2021-01-15

**Authors:** Max Eure

**Affiliations:** Lancaster University, UK

Many thanks for the time you have spent reviewing our paper and your very helpful and constructive comments on the paper. We have tried to address all of the points you have raised and to update the paper as much as is reasonably possible within the rapidly changing context. There is now an additional analysis which explores a lower (73% lower) cough or fever incidence scenario based on preliminary estimates from the Virus Watch cohort study we are running at UCL. We provide numbered point by point responses to your comments below.

**Major comments**

**Reviewer comment 1:**

 General - The authors assume that the baseline rates of fever and cough in the upcoming winter will be similar to those of previous years with additional COVID-19-related burden superimposed. Data from the Southern Hemisphere 2020 winter however suggest that this may not be the case. Most Southern Hemisphere countries did not experience an influenza season in the 2020 winter and in many countries levels of ILI consultations were at all time low levels 1. The authors should expand the abstract, introduction and discussion to consider this possibility further. It does not invalidate the relevance of the analysis but it should be clear that the projects presented may not be a true minimum estimate as it is possible that while COVID circulates other respiratory viruses may be at all-time low levels as may cases of fever and cough. The reason for the Southern Hemisphere observations are not known and it is also unclear what level of interventions will be in place to control respiratory virus transmission during the upcoming UK winter season. Hence it is unclear whether the UK will see a reduction in respiratory illness. The authors should reposition the paper to include this possibility in introduction discussion and abstract. This should also be included as a scenario in the analysis – i.e. baseline fever and cough is 50% OR 20% of historical numbers.

**Author response 1:**

 Thank you for this important comment. We have now thoroughly addressed this by discussing the possibility of low levels of other respiratory viruses and the implications of this throughout the paper and by adding an additional analysis. In this analysis we explore a low cough or fever incidence scenario (73% lower incidence than 2018-2019 levels) based on preliminary estimates from the Virus Watch cohort study we are running at UCL.

**Reviewer comment 2:**

 Methods - UK testing demand due to baseline cough and fever cases – the authors explore a range of scenarios for the proportion of cough and fever seeking testing. I think the explored ranges are reasonable but can the authors provide some context please (perhaps from other studies) on proportion of fever and cough cases which usually seek medical care (and perhaps get a sample for testing) in UK and any estimates (if they exist) or thoughts of how health seeking could increase (or decrease) in response to COVID to help contextualise which of these estimates they think more likely.

**Author response 2:**

 We have added a new reference in the discussion section which estimates  “that only about 40% of those who report classic COVID-19 symptoms go on to receive a test”. We then comment on the implications of this on COVID-19 control and our results, and stress the importance of future studies looking at test-seeking behaviour and comment on how they can be structured.

**Reviewer comment 3:**

 Methods - Could you use your study data from the summer period to look at the testing demand in the months which have already past? I.e. compare the rates for months which have past (March-August) from your study to the actual demand for testing experienced to date in the UK to see how the numbers compare and inform our thinking about how they might compare going forward. I.e. have the numbers to date been more or less than you would have predicted from your data?

**Author response 3:**

 Thank you very much for this interesting suggestion. However, given the length of the paper in its current form and to limit the number of analyses within it we have decided not to carry out this additional analysis.

**Minor comments**

**Reviewer comment 4:**

 Discussion line 3 – suggest change “will place a strain” to may place a strain.

**Author response 4:**

 We have added this suggestion.

**Reviewer comment 5:**

 Discussion – perhaps suggest some ways that UK could collect data on proportion of people with fever and cough who do get a test (e.g. through online platforms like your study) as these data may be useful to inform the public health response.

**Author response 5:**

 We have added a new reference in the discussion section which estimates  “that only about 40% of those who report classic COVID-19 symptoms go on to receive a test”. We then comment on the implications of this on COVID-19 control and our results, and stress the importance of future studies looking at test-seeking behaviour and comment on how they can be structured.

**Reviewer comment 6:**

 Discussion paragraph 5 - “Our results show that total testing demand is relatively insensitive to COVID-19 transmission” this is an interesting result – are there any published studies which have found this – perhaps again from the Southern Hemisphere – I agree this is what would be expected but it would interesting to see if this happened anywhere. COVID detection rates in different settings could be helpful but would need to know the case definitions used for testing and health seeking behaviour which data are generally not available.

**Author response 6:**

 We have been unable to find any published studies which have evaluated the sensitivity of testing demand to COVID-19 transmission and do not think we can explore this issue further in the discussion.
